# Linezolid and Its Immunomodulatory Effect: *In Vitro* and *In Vivo* Evidence

**DOI:** 10.3389/fphar.2019.01389

**Published:** 2019-11-28

**Authors:** Jin Wang, Lei Xia, Rui Wang, Yun Cai

**Affiliations:** Center of Medicine Clinical Research, Department of Pharmacy, PLA General Hospital, Beijing, China

**Keywords:** linezolid, immunomodulatory, cytokines, MAPK, inflammatory

## Abstract

Recent studies have explored the effects of some antibacterial agents on various aspects of the immune response to infection in addition to their bactericidal effects. As a synthetic oxazolidinone class of antibacterial agent, linezolid (LZD) exhibits activity against a broad range of Gram-positive bacteria. In the present review, we summarized the effects of LZD on the immune response and new approaches that can exploit such interactions for the treatment of bacterial infections. *In vitro* and pre-clinical evidence demonstrate that LZD suppresses the phagocytic ability, cytokine synthesis, and secretion of immune cells as well as the expressions of immune-related genes at the mRNA level under the stimulation of endotoxin or pathogens. Immunomodulatory effects of LZD can not only reduce the inflammatory damage induced by exaggerated or prolonged release of pro-inflammatory cytokines during infections but can also be applied to alleviate the symptoms of non-infectious inflammatory conditions. Further research is necessary to explore the molecular mechanisms involved and confirm these findings in clinical practice.

## Introduction

Infectious diseases greatly threaten human health. Several decades ago, the invention of antimicrobials brought hope to anti-infective therapy, and antimicrobials were regarded as a panacea to cure infections. Unfortunately, the accompanying antimicrobial resistance has become a great threat to humans and is a worldwide challenge associated with high morbidity and mortality. The WHO has reported that antibiotic resistance causes about 700,000 deaths each year and that this number will reach 10 million globally by 2050 if no effective intervention becomes available ((WHO) WHO, 2014).

In addition to their bacteria-targeting property, the immunomodulatory effects of some antibiotics have received increasing attention in recent years (Anuforom et al., 2015). For example, macrolide antibiotics are used in the treatment of several chronic inflammatory diseases, such as chronic obstructive pulmonary disease (COPD) (Uzun et al., 2014), asthma (Gibson et al., 2017), and non-cystic fibrosis bronchiectasis (Li et al., 2019), due to their immunomodulatory properties. Minocycline and doxycycline have shown beneficial effects on experimental colitis (Garrido-Mesa et al., 2018).

As the first synthetic oxazolidinone antimicrobial agent, linezolid (LZD) has potent activity against Gram-positive bacteria *via* the inhibition of bacterial protein synthesis through binding to rRNA (Hashemian et al., 2018). LZD has been approved for the treatment of hospital-acquired pneumonia (HAP), complicated skin and skin structure infections (SSSIs) caused by methicillin-susceptible (MSSA) or methicillin-resistant *Staphylococcus aureus* (MRSA) strains, and vancomycin-resistant *Enterococcus faecium* (VRE) infections. The overall resistance rate of LZD remains at a modest level (<1%) (Pfaller et al., 2017). Adverse effects associated with LZD include peripheral and ocular neuropathy, anemia, thrombocytopenia, hyperlactatemia, diarrhea, nausea, headache, and so on (Hashemian et al., 2018). Recently, studies have shown that LZD-containing regimens may be potential alternatives to treat patients with *multidrug-resistant* (MDR) tuberculosis (Agyeman and Ofori-Asenso, 2016). During its clinical use, although the microbiological efficacy of LZD is similar to vancomycin in most cases, it has been reported that LZD is superior to vancomycin in terms of antipyretic and anti-inflammatory effects (Yoshizawa et al., 2012). It is speculated that this finding may be attributed to the early anti-inflammatory effects of LZD. In order to comprehensively understand the research progress on LZD in immunomodulation, we systematically searched the literature in PubMed from the inception date to 30 Aug 2019 with no language restrictions. The main keywords included ‘linezolid’ OR ‘oxazolidinones’ and each of the following keywords: ‘immunomodulatory’, ‘immune’, ‘inflammatory’, ‘cytokine’, ‘chemokine’, and ‘neutrophil’. All retrieved references were imported into Endnote X9, and duplicates were discarded. The remaining articles were initially screened by reading the abstract to determine its relevance to LZD-mediated effects on the immune system. If the abstract was not sufficient for determination, the full text was read. Non-related articles were removed. The search results showed that thirty-two *in vitro* and *in vivo* studies have explored the immunomodulatory effects of LZD. All of the included references were categorized as *in vitro* studies, *in vivo* animal model studies, and clinical studies in humans. In the present review, we summarize the effects of LZD on host immune response.

### *In Vitro* Studies

#### LZD Suppresses Phagocytosis

The enhancement of phagocytosis is a hallmark of cell activation of the innate immune system. Since LZD was approved by the FDA, several studies have evaluated its effects on the phagocytosis of immune cells (**Table 1**). According to the available data, LZD shows an immunosuppressive effect since it can only suppress the phagocytic function activated by stimuli, while no obvious effect on the phagocytosis of normal immune cells has been observed. In 2003, Ballesta et al. (2003) first reported that pre-incubation of LZD at different concentrations (2, 10, and 20 mg/L) with polymorphonuclear *neutrophils* (*PMNs*) did not significantly affect the phagocytosis of PMNs against radioactively labeled *S. aureus* and *E. faecalis*. LZD at these concentrations did not affect the production of superoxide and hydrogen peroxide radicals. Naess et al. (2006) evaluated the chemotaxis, phagocytosis, and respiratory burst of human PMNs using flow cytometric techniques. They demonstrated that the pre-incubation of human PMNs with LZD at concentrations of 10–160 mg/L did not significantly affect the phagocytosis and respiratory burst of human PMNs. Chemotaxis was only slightly increased after incubation with LZD. Although these studies indicated that LZD had no direct effect on phagocytosis, it could suppress the phagocytic ability activated by lipopolysaccharide (LPS). Bode et al. (2015) found that LZD significantly inhibited phagocytotic activity against heat-killed *E. coli* by 25% compared with LPS-activated controls in THP-1 monocytes.

**Table 1:** *In vitro* studies of the effect of LZD on the immune response.

Author	Year	*In vitro* model	Stimulants or pathogens	Immune response
(Ballesta et al., 2003)	2003	Human PMN	MRSA, MSSA, VSE, VRE	Preincubation of PMN with different concentrations of LZD (2, 10, and 20 mg/L) neither affects the phagocytosis of PMN against strains nor affects its production of superoxide and hydrogen peroxidase radicals.
(Kushiya et al., 2005)	2005	human PBMCs	1ng/ml of TSST-1	LZD (10 and 40 µg/mL) had no significant inhibitory effect on cytokine production, which only increased TNF-a by 1.3 ± 16.2% and 18.1 ± 10.6%.
(Naess et al., 2006)	2006	Human PMN	Rhodamine green X-labeled *S. aureus*	LZD at concentrations of 10-160 mg/L did not significantly influence PMN function as measured by chemotaxis, phagocytosis, and respiratory burst.
(Garcia-Roca et al., 2006)	2006	PBMCs	100 ng/mL of LPS	LZD (1, 5, 10, and 30 µg/mL) significantly suppressed the synthesis of the IL-1β, IL-6, TNF-a, and IL-1ra in a concentration-dependent manner.
(Takahashi et al., 2010)	2010	Human whole blood	10 µg/mL of LPS	Significant decreases in TNF-α and IFN-γ were observed in the LZD 2, 4, and 15 µg/ml groups compared with the no LZD treatment group. No significant decrease of the endotoxin level was observed in the LZD 2, 4, or 15 µg/ml groups as compared with the no LZD treatment group.
(Lambers et al., 2010)	2010	Human whole blood	50 pg/mL LPS	LZD at 13 μg/mL significantly reduced mRNA levels of IL-1β, IL-6, IL-8, and TNF-alpha after 2 and 4 h. However, except for IL-6, no significant reduction at the protein level was observed.
(Pichereau et al., 2012)	2012	Human PBMCs	100 ng/mL TSST-1, SEA, α-toxin or PVL	25 mg/L LZD inhibited TNF-α by 12%-35% and IL-8 by 25%-42% (P ≤ 0.02) compared with toxin alone.
(Franks et al., 2013)	2013	Human whole blood	MRSA	MRSA accelerated thrombin generation and induced the release of cytokines, including IL-6, IL-8, and MCP-1. Early administration of 5.0 μg/mL LZD restored normal thrombin generation patterns and significantly reduced the synthesis of cytokines.
(Kaku et al., 2014)	2014	Human airway epithelial cells	MRSA	LZD significantly reduced MRSA-induced MUC5AC protein and mRNA overexpression at concentrations of 5 and 20 μg/mL. ERK1/2 was phosphorylated by the MRSA supernatant. ERK1/2 phosphorylation was inhibited by LZD.
(Bode et al., 2015)	2015	Human monocytic THP-1 cells	10 µg/mL LPS	20 µg/mL LZD greatly increased the mRNA expression of TNF-α, IL-1β, and IL-6 compared with unstimulated controls at 6 and 24 h, whereas IL-10 was significantly reduced at 24 h. Mono-stimulation of cells with linezoid significantly increased the gene expression of TLR. LZD in combination with LPS significantly reduced phagocytotic activity by 25% compared with LPS-activated controls.
(Chen et al., 2015)	2015	Murine macrophages	100 ng/ml LPS	A series of novel oxazolidinone compounds designed and synthesized using LZD as a lead exhibited significant inhibitory activities on the production of inflammatory mediators including NO, IL-6, and TNF-α.

MSSA, methicillin-susceptible S. aureus; MRSA, methicillin-resistant S. aureus; GISA, glycopeptides intermediate S. aureus; VSE, vancomycin-susceptible E. faecalis; VRE, vancomycin-resistant E. faecalis; TSST-1, toxic shock syndrome toxin-1; SEA, staphylococcal enterotoxin A, PVL, Panton-Valentine leucocidin; PMN, polymorphonuclear neutrophils; PBMCs, peripheral blood mononuclear cells; MCP-1, monocyte chemotactic protein -1.

#### LZD Suppresses Cytokine Secretion

Cytokines are essential mediators in regulating the immune response during infection. Cytokines can be divided into pro-inflammatory and anti-inflammatory cytokines (Schulte et al., 2013). Under normal conditions, pro-inflammatory cytokines act as crucial signals in the development of appropriate defenses. However, exaggerated or prolonged release can lead to pathological conditions. For example, during sepsis, excessive production of pro-inflammatory cytokines induced by bacterial toxins has been indicated to be responsible for mortality (Eichacker et al., 2002). Several of the studies included in the current review reported that LZD can suppress the synthesis of pro-inflammatory cytokines, such as interleukin-1β (IL-1β), IL-6, IL-8, interferon-γ (IFN-γ), and tumor necrosis factor-α (TNF-α) (**Table 1**). Generally speaking, LZD exhibits no direct effect on cytokine synthesis. However, it can suppress the cytokine production induced by different cytotoxins produced by pathogens.

Toxic shock syndrome toxin-1 (TSST-1), staphylococcal enterotoxin A (SEA), a-toxin, and Panton-Valentine leucocidin (PVL) are potent cytotoxins produced by highly virulent *S. aureus* strains, which can induce leukocyte activation and cytokine overproduction. Kushiya et al. (2005) compared the inhibitory effects of several antibiotics on TSST-1-induced cytokine production of peripheral blood mononuclear cells (PBMCs). They found that LZD led to a reduction of 18.1 ± 10.6% in TNF-α production, although the difference was not significant compared with the control group (p > 0.05). However, Pichereau et al. (2012) reported that 25 mg/L LZD could decrease the TNF-α concentration of human PBMCs by 35%, 12%, 24%, and 36% and that such a dose could decrease the IL-8 concentration by 25%, 32%, 35%, and 42% after exposure to PVL, TSST-1, SEA, and a-toxin, respectively. LZD exerted its inhibitory effect in a concentration-dependent manner within the concentration range of 5-100 mg/L. (Pichereau et al., 2012).

LPS, which exists in the outer membrane of Gram-negative bacteria, can induce cytokine release. A study using PBMCs donated by volunteers revealed that LZD (1, 5, 10, and 30 µg/mL) could suppress the synthesis of IL-1β, IL-6, TNF-α, and IL-1ra in a concentration-dependent manner. Generally, LZD strongly inhibited the synthesis of cytokines within a concentration range of 10-30 µg/mL (Garcia-Roca et al., 2006). A similar inhibitory effect of LZD has been found in LPS-stimulated cytokine production in peripheral venous whole-blood. The levels of TNF-α and IFN-γ were significantly decreased in 2, 4, and 15 µg/mL LZD groups compared with controls. The levels of IL-10, monocyte chemotactic protein (MCP)-1, and endotoxin were not significantly decreased in the LZD groups. Therefore, that study concluded that LZD suppresses the production of TNF-α and IFN-γ without exerting any inhibitory effect on endotoxin production by bacteria (Takahashi et al., 2010). (Chen et al., 2015) compared the effects of a series of novel oxazolidinone compounds and LZD on the production of inflammatory mediators (NO, IL-6, and TNF-α) in LPS-stimulated murine macrophages. They found that some of these oxazolidinone compounds exhibited a more significant inhibitory effect than LZD.

Franks et al. (2013) developed a human whole blood model to evaluate the benefits of early antibiotic administration in reducing the MRSA-induced thrombo-inflammatory “cytokine storm.” LZD and vancomycin both suppressed the synthesis of IL-6, IL-8, and MCP-1 when they were added within 3 h of MRSA inoculation. LZD alone significantly reduced the cytokine synthesis within 6 h of MRSA inoculation compared with vancomycin. When the antibiotic administration was postponed to 9 h post MRSA inoculation, neither LZD nor vancomycin significantly reduced cytokine production compared with antibiotic-free MRSA inoculates.

#### Effect of LZD on Gene Expression

Lambers et al. (2010) investigated the immunomodulatory effects of LZD at the molecular level in human whole blood. Blood samples collected from volunteers were incubated either with 50 pg/mL LPS and saline or 50 pg/mL LPS plus 13 µg/mL LZD. The addition of LZD significantly decreased the expressions of IL-1β, IL-6, IL-8, and TNF-α at the mRNA level after 2 h and 4 h. Meanwhile, the addition of LZD alone resulted in a significant reduction of IL-6 after 2 h, while the levels of TNF-α and IL-8 were barely changed. Therefore, they speculated that LZD exerted the immunomodulatory effect mainly through down-regulating the pro-inflammatory cytokines rather than by inhibiting cytokine release.

Nevertheless, the expressions of cytokines at the mRNA level showed a different pattern in human monocytic THP-1 cells (Bode et al., 2015). In the absence of LPS, LZD alone greatly increased the expressions of TNF-α, IL-1β, and IL-6 at the mRNA level compared with unstimulated controls at 6 h and 24 h, whereas the expression of IL-10 was significantly reduced at 24 h. In the presence of LPS, LZD still significantly up-regulated the expressions of TNF-α, IL-6, and IL-1β at different time points. Unlike in the absence of LPS, the expression of IL-10 was up-regulated by LZD in combination with LPS at all investigated time points. Toll-like receptors (TLRs) play an outstanding role in the primary recognition of microorganisms. TLRs activate signaling cascades, leading to the induction of pro-inflammatory cytokines. In this study, the mono-stimulation of cells with LZD significantly increased the expressions of TLR1, 2, 6, and 9 compared with unstimulated controls at 24 h. When LZD was co-administered to LPS-activated cells, the highest expressions of TLRs were observed after 24 h of incubation, and all investigated TLRs (TLR1, 2, 4, 6, 7, and 9) were up-regulated compared with LPS-activated controls. However, the study did not provide data to confirm the promotive effect of LZD on the cytokines at the protein level. The difference might be attributed to the different cell types. Previous studies investigated the effects of LZD on whole blood samples, such as PBMCs, while this study only focused on monocytic THP-1 cells.

Mucin is an important barrier in airway epithelium due to its ability to trap inhaled microbial organisms, particulates, and foreign irritants. MUC5AC is a gel-forming mucin that is strongly expressed in the lung (Kirkham et al., 2002). However, mucin over-expression causes many problems, such as airway obstruction, atelectasis, impaired oxygenation, and reduced antibiotic permeability. MUC5AC over-expression has been observed in patients with chronic respiratory diseases and ventilator-associated pneumonia (VAP) (Dennesen et al., 2003; Williams et al., 2006). Therefore, inhibition of MUC5AC over-expression seems to be useful. Kaku et al. (2014) examined the effect of LZD on MRSA-induced MUC5AC over-expression in airway epithelial cells. LZD significantly reduced MRSA supernatant-induced MUC5AC protein production and mRNA expression at concentrations of 5 and 20 µg/mL. Furthermore, MUC5AC over-expression was caused by the activation of a MAPK pathway member, ERK1/2, while LZD inhibited the phosphorylation of ERK1/2.

### *In Vivo* Studies

#### Murine Model of MRSA-Induced Pulmonary Infection

Pneumonia is one of the main indications for LZD (**Table 2**). Therefore, the immunomodulatory effects of LZD have also been intensively studied in the murine pneumonia model. Overall, almost all studies have demonstrated that LZD can effectively reduce the concentration of pro-inflammatory cytokines in tissue or bronchoalveolar lavage fluid (BALF) and ameliorate the tissue inflammatory damage, while its antibacterial efficacy is similar to other antibiotics for Gram-positive bacteria. Yanagihara et al. (2002) first observed that LZD significantly improved survival and decreased inflammatory damage in lung tissue compared with vancomycin and teicoplanin in an immunocompromised mouse model of vancomycin intermediate *S. aureus* (VISA) hematogenous pulmonary infection. Next, they set up a mouse model of haematogenous pulmonary infection caused by PVL-positive *S. aureus*. Similar to their previous results, the number of viable bacteria in the lungs of LZD-treated mice was significantly lower compared with the vancomycin group and the survival rate was higher than that of the vancomycin group (Yanagihara et al., 2009). Moreover, the LZD group exhibited fewer abscesses and less inflammation than the control and vancomycin groups. The concentrations of pro-inflammatory cytokines (TNF-α, MIP-2, and IL-1β) in lung homogenates were significantly lower in both the vancomycin and LZD groups compared with the control group. Although the difference was not significant, the cytokine concentrations were numerically lower in the LZD group compared with the vancomycin group.

**Table 2:** Animal model studies of the effect of LZD on the immune response.

Author	Year	Animal model types	Stimulants or pathogens	Immune response
Yanagihara et al., 2002	2002	Mouse model of pulmonary infection	MRSA or VISA	LZD (100 mg/kg/day) significantly improved survival and decreased inflammatory damage in lung tissue compared with vancomycin and teicoplanin.
Yanagihara et al., 2009	2009	Mouse model of pulmonary infection	PVL-positive *S. aureus*	Histopathological examination showed the beneficial efficacy of LZD (100 mg/kg/dose, bid) compared with vancomycin (100 mg/kg/dose, bid). Concentrations of TNF-α, MIP-2, and IL-1β were significantly lower in both the treatment groups compared with control. All of the cytokine concentrations were numerically lower in the LZD group than in the vancomycin group.
Luna et al., 2009	2009	Piglet model of mechanically ventilated pneumonia	MRSA	LZD (300 mg q8h), vancomycin (500 mg q6h), and teicoplanin (200 mg q12h) resulted in a significantly enhanced survival rate and decreased CRP in serum and cytokines (TNF-α and IL-6) in lung fluid, but no difference was noted among the antibiotics.
Akinnusi et al., 2011	2011	Mouse model of pneumonia	MRSA	No difference in BAL of monocyte chemotactic protein-5, IL-6, matrix metalloproteinase-9, and neutrophil apoptosis between LZD (80 mg/kg q12 h)- and vancomycin (110 mg/kg q12 h)-treated groups.
Yoshizawa et al., 2012	2012	Mouse model of pneumonia	MRSA	LZD (0.4 mg/mouse; 12 mg/kg) but not vancomycin (1 mg/mouse; 40 mg/kg) treatment significantly reduced induction of inflammatory cytokines in the lungs. Sub-MICs of LZD revealed significant suppression of IL-6 in a dose-dependent manner, but pretreatment of mice with LZD resulted in no changes in cytokines.
Martinez-Olondris et al., 2012	2012	Piglet model of mechanically ventilated pneumonia	PVL-negative MRSA	Severe inflammation was only significantly reduced in the LZD (15 mg/kg q12h) group. No differences were found between LZD and vancomycin (10 mg/kg q12h or 1 g continuous infusion with an initial bolus of 250 mg over 60 minutes) groups of serum TNF-α, IL-6, and IL-8 levels determined 12 h after inoculum and at the end of the study.
Breslow-Deckman et al., 2013	2013	Mouse model of post-influenza bacterial pneumonia	*Streptococcus pneumoniae*	LZD (20 mg/kg, 40 mg/kg, or 80 mg/kg q12h) pretreatment led to decreased IFN-γ and TNF-α production, decreased weight loss, and lower bacterial burdens at 24 h post bacterial infection in comparison with vehicle-treated controls.
Liu et al., 2013	2013	Mouse model of post-influenza bacterial pneumonia	Influenza A virus and 3 days later with MRSA	LZD (100 mg/kg q12 h), vancomycin (180 mg/kg q12h), and clindamycin (300 mg/kg q8h) significantly decreased pulmonary IL-6 and mKC at 4 h and IFN-γ at 24 h after MRSA coinfection, while IL-1β, TNF-α, and IL-12 were similar to placebo groups. LZD and clindamycin, but not vancomycin, significantly decreased IL-10 at 4 h after MRSA infection.
Chen et al., 2013	2013	Mouse model of pneumonia	MRSA	LZD (100 mg/kg/day) significantly decreased protein concentration and levels of cytokines including IL-6, IL-1β, Interferon-γ, and IL-17 in BAL. No significant gene expression changes in the mouse lungs were associated with LZD therapy.
Al-Banna et al., 2013	2013	Rats with CASP-induced sepsis or after endotoxin challenge	LPS	Single-administration LZD (25 mg/kg) increased the intestinal FCD, which was reduced during CASP-induced sepsis. The number of adherent leukocytes increased 3-fold in rats with CASP sepsis. It was reduced following administration of LZD. Administration of tigecycline (5 mg/kg) and LZD reduced the LPS-induced increase in the number of adherent leukocytes by 50%.
Sharma-Kuinkel et al., 2013	2013	Murine sepsis model	*S. aureus*	PVL, IL-1β, and IL-6 were significantly reduced in LZD (25 mg/kg)-treated mice compared to untreated mice. Neither treatment significantly reduced the production of TNF-α. Expression of immunomodulatory genes like Cxcl9, Cxcl10, Il1r2, Cd14, and Nfkbia was different among the LZD and vancomycin treatment groups. The glycerolipid metabolism pathway was uniquely associated with LZD treatment in *S. aureus* infection.
Diep et al., 2013	2013	Rabbit model of necrotizing pneumonia	MRSA	Compared with untreated and vancomycin-treated (30 mg/kg q12h) rabbits, improved survival of rabbits with LZD (50 mg/kg q8h) was associated with a significant decrease in bacterial counts, bacterial production of PVL and Hla, and IL-8 in the lungs.
Cabellos et al., 2014	2014	Rabbit model of meningitis	GISA	LZD (20 mg/kg q4h) and its combination with a rifampicin (15 mg/kg q24h) strain improved the levels of the inflammatory parameters lactate and protein in CSF compared with vancomycin and control.
Jacqueline et al., 2014	2014	Mouse model of pneumonia	MRSA	LZD (80 mg/kg q12h) dramatically reduced the production of TNF-α and neutrophil infiltration in infected lung tissues compared with vancomycin (110 mg/kg q12h).
Matsumoto et al., 2015	2015	Rat model of Carrageenan-induced paw edema	Carrageenan	Pretreatment of LZD (50 mg/kg) significantly suppressed edema rates compared with control and vancomycin (50 mg/kg), teicoplanin (50 mg/kg), arbekacin (50 mg/kg), and daptomycin (50 mg/kg). LZD exhibited anti-inflammatory activity in a concentration-dependent manner from 5 to 50 mg/kg.
Bhan et al., 2015	2015	Murine model of post influenza bacterial pneumonia	MRSA	The LZD (80 mg/kg) group had significantly lower numbers of neutrophils in the BAL, associated with reduced levels of chemotactic chemokines and inflammatory cytokines KC, MIP-2, IFN-γ, TNF-α, and IL-1β in the BAL compared with vancomycin (110 mg/kg). LZD treatment protected mice from lung injury, as assessed by the albumin concentration in the BAL post treatment with H1N1 and cMRSA when compared to vancomycin treatment. Moreover, treatment with LZD was associated with significantly lower levels of PVL toxin in the lungs.
Kaur et al., 2016	2016	Murine model of joint infection	MRSA	An LZD-coated implant showed significantly low levels of IL-1β and TNF-α on all days as compared to untreated mice. The degree of infiltration of lymphocytes and plasma cells was comparatively limited in the LZD coated wire group.
Kaku et al., 2016	2016	Murine model of pulmonary infection	MRSA	LZD (120 mg/kg q12h) and tedizolid (20 mg/kg q24h)) significantly decreased the plasma concentrations of TNF-α, IL-6, and MIP-2 in comparison with the control and vancomycin groups.
Pauchard et al., 2017	2017	Rabbit model of spontaneously breathing or mechanically ventilated pneumonia	MRSA	Both treatment with LZD (50 mg/kg q12h) alone and with statin (20 mg/kg) alone significantly reduced lung TNF-α concentrations in both spontaneously breathing and mechanically ventilated groups but not the IL-8 level. Prior statin treatment alone and a combination of statin and LZD significantly decreased IL-8 in blood and spleen in mechanically ventilated animals, whereas LZD alone did not.
Verma et al., 2019	2019	Murine model of post influenza bacterial pneumonia	MRSA	Mice that received LZD (50 mg/kg/day) and gentamicin (100 mg/kg, followed by150 mg/kg/day) had significantly reduced TNF-α and IL-6 levels in BAL compared to vancomycin-treated (300 mg/kg, followed by 150 mg/kg/day) counterparts during MRSA and influenza coinfection. Moreover, LZD significantly reduced the levels of albumin and LDH activity in BAL.

MRSA, methicillin-resistant S. aureus; GISA, glycopeptides intermediate S. aureus; CRP, C-reactive protein; BAL, bronchoalveolar lavage; CASP, colon ascendens stent peritonitis; FCD, intestinal functional capillary density; PVL, Panton-Valentine leucocidin; MIP, macrophage-inflammatory protein; CSF, cerebro-spinal fluid.

A number of studies have subsequently explored the effects of LZD on the expressions of cytokines in lung tissue homogenates, BALF, or plasma using a murine model of MRSA pneumonia. Akinnusi et al. (2011) did not observe a significant difference in IL-6 or MCP-1 concentration in BALF between LZD- and vancomycin-treated mice. Chen et al. (2013) found that LZD therapy significantly decreased BALF protein concentration and levels of cytokines, including IL-6, IL-1β, IFN-γ, and IL-17. With LZD treatment, no abscesses formed, less lung edema occurred, and fewer inflammatory cells were observed compared with the control group. No significant changes of gene expressions in the mouse lungs were associated with LZD therapy. Cytokine levels in the lungs after MRSA inoculation were determined in a study by Yoshizawa et al. (2012). The levels of IL-6, IL-12, and TNF-α in the lung homogenates were significantly reduced by LZD administration but not by vancomycin. TNF-α and IL-6 in the lung were significantly down-regulated by LZD administration in a dose-dependent manner. Jacqueline et al. (2014) showed that LZD and vancomycin have similar antibacterial activity in MRSA-induced pneumonia. However, LZD alone was able to reduce the production of TNF-α in lung homogenates dramatically. Analyses of myeloperoxidase activity and Ly6G immunostaining also showed a dramatic decrease in neutrophil infiltration in infected lung tissues of LZD-treated animals. Kaku et al. (2016) investigated the immunomodulatory effects of tedizolid, LZD, and vancomycin by evaluating the concentrations of inflammatory cytokines in plasma. Although there were no significant differences in the bacterial count in the lungs among the three antibiotics, tedizolid or LZD alone significantly decreased the plasma concentrations of TNF-α, IL-6, and MIP-2.

#### Murine Model of Post-Influenza Bacterial Pneumonia

Post-influenza *S. aureus* pneumonia was identified as a common cause of death during the recent H1N1 influenza pandemic (**Table 2**). A recent study showed that co-infection with influenza and MRSA led to increased inflammation and more severe lung injury compared with MRSA alone (Lee et al., 2010). The four studies included all showed that LZD can decrease the production of pro-inflammatory cytokines and reduce inflammatory injury of the lung.

Liu et al. (2013) established a murine model of moderately severe influenza MRSA co-infection and compared the efficacy of LZD, vancomycin, and clindamycin on bacterial and viral titers, as well as pulmonary cytokines. Antibiotic-treated mice had lower CFU of MRSA in the lungs compared with placebo at 4 h and 24 h after MRSA inoculation. No significant differences in the colony counts of MRSA and influenza viral titers were found among the three antibiotics. LZD mono-treatment showed a 1-log lower PFU compared with the other treatment groups at 4 h after MRSA infection. However, the difference was not statistically significant. The levels of TNF-α, IL-1β, IL-6, IL-10, IL-12, mKC, and IFN-γ were significantly increased at 4 h or/and 24 h after MRSA co-infection. LZD, vancomycin, or clindamycin significantly decreased pulmonary IL-6 and mKC levels at 4 h and the IFN-γ level at 24 h after MRSA co-infection, while the levels of IL-1β, TNF-α, and IL-12 were similar to those of the placebo group. LZD and clindamycin, but not vancomycin, were associated with a decreased concentration of IL-10 at 4 h after MRSA infection. Basically, LZD showed a similar effect on cytokines as clindamycin. Breslow-Deckman et al. (2013) demonstrated that an oral dosage of 20 mg/kg, 40 mg/kg, or 80 mg/kg of LZD twice daily sufficiently decreased the IFN-γ level in BALF at day 7 post-influenza infection in a dose-dependent manner. When mice were intranasally challenged with *S. pneumoniae* at 7 days after influenza infection, LZD pre-treatment also decreased IFN-γ and TNF-α production and weight loss, showing lower bacterial burdens at 24 h post bacterial infection compared with controls. Intranasal instillations of recombinant IFN-γ to LZD-treated animals before *S. pneumonia* challenge could partially reverse the protective effects observed in the LZD-treated mice. Therefore, they speculated that the modulatory effects of LZD were partially mediated by its ability to blunt IFN-γ production. Bhan et al. (2015) found that LZD was as effective as vancomycin in reducing bacterial burden in the lungs of mice infected with influenza followed by cMRSA. However, the numbers of neutrophils, chemotactic chemokines (KC/CXCL1 and MIP-2/CXCL2), and pro-inflammatory cytokines (TNF-α, IFN-γ, and IL-1β) in the BALF of the LZD group were significantly lower than in the vancomycin group. Albumin in the BALF of the LZD group was significantly decreased, indicating that LZD treatment can protect mice from lung injury. The most recent study published in 2019 (Verma et al., 2019) showed that LZD therapy significantly improved animal survival from post-influenza MRSA pneumonia as compared with vancomycin treatment. Rather than improved viral or bacterial control, this advantageous therapeutic effect was associated with significantly attenuated pro-inflammatory cytokine response (TNF-α and IL-6) and acute lung damage in LZD-treated mice. Gentamicin could also significantly reduce TNF-α, IL-6, albumin, and LDH activity in BAL during MRSA and influenza coinfection.

#### Mechanical Ventilation-Associated Pneumonia (VAP) Model

VAP is the first cause of mortality among nosocomial infections. MRSA is a common pathogen of VAP. The main findings from animal VAP models are that treatment with LZD leads to a better microbiological and histopathological response than do other antibiotics. Two studies have evaluated the LZD efficacy with a piglet model of mechanical VAP infected with MRSA (**Table 2**). One study (Luna et al., 2009) revealed that LZD, vancomycin, or teicoplanin resulted in a significantly enhanced survival rate and decreased C-reactive protein (CRP) in serum and cytokine production (TNF-α and IL-6) in lung fluid, while no difference was noted among the antibiotics. However, the MRSA-negative rate in blood cultures or lung fluids was significantly reduced in the LZD group compared with the other treatment groups. Martinez-Olondris et al. (2012) compared the effects of LZD (15 mg/kg) with two different dosages of vancomycin (10 mg/kg twice daily or continuous infusion with an initial bolus of 250 mg in 1 h) in mechanically ventilated piglets infected with PVL-negative MRSA. Although all treatments significantly reduced MRSA-positive cultures in BALF specimens, only LZD and high-dose vancomycin significantly reduced MRSA-positive cultures from lung tissues. All treatments demonstrated histopathological relief of inflammation in lung tissue. However, severe inflammation was only significantly reduced in the LZD group. No differences were found in the serum levels of TNF-α, IL-6, and IL-8 between groups at 12 h after inoculation and at the end of the study.

Pauchard et al. (2017) compared LZD alone, atorvastatin alone, and their combination in both spontaneously breathing and mechanically ventilated rabbit pneumonia models. Both LZD and statin mono-treatments significantly reduced lung TNF-α concentrations in both the spontaneously breathing and mechanically ventilated groups but not IL-8. Pretreatment with statin alone and the combination of statin and LZD significantly decreased the IL-8 level in blood and spleen in mechanically ventilated animals, whereas no such effect was observed in the LZD-alone treatment. However, the combination of LZD and statin led to an increased rate of bacteremia in mechanically ventilated animals, which might be attributable to the dampened systemic inflammatory response hampering blood defenses against MRSA.

#### Murine Sepsis Model

Different sepsis models have been established. LZD exerts an inflammatory response-reducing effect, and tigecycline and vancomycin show similar effects. Al-Banna et al. (2013) applied two experimental sepsis models, a colon ascendens stent peritonitis (CASP)-induced sepsis rat model (a plastic stent is inserted in the intestinal wall, leading to continuous faecal outflow into the abdominal cavity and causing peritonitis and sepsis) and a LPS-induced endotoxemia rat model, to study the impact of seven antibiotics relevant to clinical sepsis on intestinal leukocyte recruitment and capillary perfusion. The results showed that a single administration of LZD, tigecycline, or daptomycin could increase the intestinal functional capillary density, which was decreased during CASP sepsis. LZD, tigecycline, and daptomycin could significantly reduce the number of adherent leukocytes, which was increased several- fold in rats with CASP sepsis. In the LPS-induced endotoxemia model, tigecycline and LZD also reduced the increase in the number of adherent leukocytes by 50%. The results indicated the beneficial effects of LZD, erythromycin, tigecycline, and daptomycin of improving intestinal capillary perfusion and/or reducing leukocyte recruitment. Sharma-Kuinkel et al. (2013) intraperitoneally injected 6×10^6^ CFU/g of MRSA into mice. Bacteria were demonstrable in the murine bloodstream within 2 h post infection, mimicking sepsis. Both LZD and vancomycin treatments were associated with significantly reduced PVL production compared with the control group. However, LZD and vancomycin did not significantly differ in their reduction of PVL production. Both antibiotics significantly reduced IL-1β production in serum compared with the untreated group, while the difference between LZD and vancomycin was not significant. LZD treatment alone resulted in a statistically significant reduction in IL-6 production compared with the untreated controls. Neither LZD nor vancomycin treatment resulted in a statistically significant reduction of TNF-α production. Whole-blood gene expression profiling showed that LZD alters the expressions of a greater number of genes in infected mice compared with vancomycin, including immunomodulatory genes like Cxcl9, Cxcl10, Il1r2, Cd14, and Nfkbia. The superiority of LZD may not only be attributable to its better effect on lowering the *in vivo* levels of toxins but also to its better immunomodulatory effects.

#### Other Animal Models

A rabbit model of MRSA-induced necrotizing pneumonia has been used to compare the therapeutic effects of LZD and vancomycin (Diep et al., 2013). Early treatment (1.5 h after infection) with 50 mg/kg LZD was associated with a significantly improved survival rate as well as decreased bacterial counts and PVL, α-hemolysin (Hla), and IL-8 levels in lung homogenates compared with the control and vancomycin (30 mg/kg) groups. Since the correlation between levels of PVL and IL-8 in lungs from LZD-treated rabbits was strong, the effects of LZD on host inflammatory response were partially attributed to the inhibited production of potent bacterial toxins like PVL.

Cabellos et al. (2014) established a meningitis model induced by injecting a glycopeptide intermediate *S. aureus* (GISA) strain into the rabbit cisterna magna. Four treatment regimens, including a control group (saline), LZD, vancomycin, and LZD plus rifampicin, were applied. At 24 h after therapy, levels of inflammatory parameters (lactate and protein) in the cerebro-spinal fluid (CSF) from the LZD group were significantly lower compared with the other treatments.

A recent study compared the anti-inflammatory activities of LZD and other anti-MRSA agents (vancomycin, teicoplanin, arbekacin, and daptomycin) using the carrageenan-induced rat paw edema model (Matsumoto et al., 2015). Carrageenan is a strong chemical that is used to stimulate the release of inflammatory and pro-inflammatory mediators. Pretreatment with 50 mg/kg LZD significantly suppressed edema rates compared with the 5% glucose group. Moreover, this effect was found to occur in a dose-dependent manner at doses of 5, 10, 25, and 50 mg/kg. Edema rates were not decreased in pretreatments with 50 mg/kg vancomycin, teicoplanin, arbekacin, and daptomycin.

A local drug delivery system is used as a strategy to kill pathogenic bacteria by delivering a high drug concentration at the implant site in orthopedic implant infections. Kaur et al. (2016) evaluated the efficacy of dual coated biodegradable polymer K-wires impregnated with both lytic phage and LZD in a murine model of experimental joint infection. Cytokine levels of IL-1β and TNF-α were significantly decreased in phage alone, LZD alone, and dual coated groups compared with the untreated mice, while mice implanted with dual coated wire showed minimum levels of IL-1β and TNF-α at all time points.

#### Clinical Studies in Humans

Compared with *in vitro* and animal model studies, the immunomodulatory effect of LZD is much less clinically studied in humans. So far, there have been only two clinical reports. A retrospective study including 52 patients with MRSA infections was undertaken to investigate the prompt defervesce effect of LZD (Yoshizawa et al., 2012). The results showed that 64% of febrile patients demonstrated significant defervescence within 3 days despite having positive culture results. The median time of defervescence (3 days) is significantly shorter than that under the culture-negative condition (8 days). A randomized clinical trial was designed to explore the effect of LZD on cytokine levels from extracts of inflammatory periapical tissues (Danin et al., 2003). A total of 22 patients with root-filled teeth and persistent periapical pathosis were randomly divided into an LZD group and a control group. After continuous administration of LZD (600 mg) for 5 days, periapical tissue was collected from the root-end of one tooth from each patient. IL-1ra, IL-6, and transforming growth factor beta (TGF-β) levels were determined. IL-1ra was significantly decreased in the LZD group compared with the control group, while IL-6 and TGF-β remained unchanged. The researchers did not suggest the use of LZD for the treatment of periapical infections because it should be reserved for MDR pathogens, albeit the results implied that LZD attenuates the severity of inflammation in periapical tissues.

## Conclusions

Since LZD was approved for clinical use in 2002, an increasing amount of evidence has supported the possibility that LZD can affect the immune response. As indicated by the *in vitro* and *in vivo* evidence summarized in this review, the available data demonstrate that LZD suppresses the phagocytic ability, cytokine synthesis, and secretion of immune cells under the stimulation of endotoxin or pathogens. The effect of LZD on immune-related genes has also been initially investigated at the mRNA level. These works enrich our knowledge of the impact of LZD during clinical use. On the one hand, the immunomodulatory properties of LZD can be used to benefit bacterial infections. By down-regulating inflammatory cascade, LZD can reduce the inflammatory damage induced by exaggerated or prolonged release of pro-inflammatory cytokines during critical infections. On the other hand, because the immunomodulatory effect of LZD is distinct from its antibacterial effects, LZD can also be applied to alleviate the symptoms of noninfectious inflammatory conditions, such as ulcerative colitis.

Some issues still need to be addressed in future studies. First of all, although studies using cells, tissue cultures, and animal models have provided a good platform for investigating the effect of LZD on host immune status and the outcome of antibacterial therapy, more studies are required to explore the molecular mechanisms and confirm those findings in clinical practice. Second, it is necessary to evaluate the risk of increasing antibiotic resistance when using antibiotics, such as LZD, in patient populations that are likely infected with bacteria (cystic fibrosis, non-cystic fibrosis bronchiectasis, COPD). Finally, the finding that LZD exerts a cytokine-inhibiting effect means that it will exhibit an immunoinhibitory effect. Therefore, the potential negative effect of LZD administration in immunosuppressed patients must be considered.

In conclusion, the present review suggests that the immunomodulatory activities of LZD have a protective effect against destructive local inflammatory responses in areas of infection. Antibiotics that have potential effects on the immune response are worth exploiting further while considering the above-mentioned issues.

## Author Contributions

JW and LX contributed in data collection, analysis and writing the paper. RW contributed in study design and analysis. As a corresponding author, YC contributed in study design, protocol, data analysis and writing.

## Funding

This research was supported by the National Natural Science Foundation of China (No. 81573472 and No.81770004) and 13th Five-Year Plan of National Major Science and Technology Projects of China (No. 2018ZX09201-013).

## Conflict of Interest

The authors declare that the research was conducted in the absence of any commercial or financial relationships that could be construed as a potential conflict of interest.
